# Serum uric acid levels in patients with Parkinson’s disease: A meta-analysis

**DOI:** 10.1371/journal.pone.0173731

**Published:** 2017-03-20

**Authors:** Min Wen, Bo Zhou, Yun-Hua Chen, Zhao-Lei Ma, Yun Gou, Chun-Lin Zhang, Wen-Feng Yu, Ling Jiao

**Affiliations:** 1 Department of Anatomy, Guizhou Medical University, Guiyang, China; 2 Department of Biology, Guizhou Medical University, Guiyang, China; 3 Department of Neurology, BaiYun Hospital, Guizhou Medical University, Guiyang, China; 4 Key Laboratory of Endemic and Ethnic Diseases, Ministry of Education, P. R. China; Key Laboratory of Medical Molecular Biology, Guizhou Medical University, Guizhou Province, Guiyang, P. R. China; 5 Department of Neurology, Affiliated Hospital, Guizhou Medical University, Guiyang, China; Leibniz-Institut fur Pflanzengenetik und Kulturpflanzenforschung Gatersleben, GERMANY

## Abstract

**Background:**

Lower serum uric acid (UA) levels have been reported as a risk factor in Parkinson’s disease (PD). However, the results have been inconsistent so far.

**Objectives:**

The aim of the present study was to clarify the potential relationship of uric acid with PD.

**Methods:**

Comprehensive electronic search in pubmed, web of science, and the Cochrane Library database to find original articles about the association between PD and serum uric acid levels published before Dec 2015. Literature quality assessment was performed with the Newcastle-Ottawa Scale. Random-effects model was used to estimate the standardized mean differences (SMDs) with 95% confidence intervals (CIs). Heterogeneity across studies was assessed using I^2^ and H^2^ statistics. Sensitivity analyses to assess the influence of individual studies on the pooled estimate. Publication bias was investigated using funnel plots and Egger’s regression test. Analyses were performed by using Review Manager 5.3 and Stata 11.0.

**Results:**

Thirteen studies with a total of 4646 participants (2379 PD patients and 2267 controls) were included in this meta-analysis. The current results showed that the serum UA levels in PD patients were significantly lower compared to sex and age-matched healthy controls (SMD: -0.49, 95% CI: [-0.67, -0.30], *Z* = 5.20, *P* < 0.001) and these results showed no geographic regional (Asia: SMD = −0.65, 95% CI [−0.84, −0.46], Z = 6.75, *p* <0.001; Non-Asia: SMD = −0.25, 95% CI [−0.43, −0.07], Z = 2.70, *p* = 0.007) and sex differences (women: SMD = −0.53, 95% CI [−0.70, −0.35], z = 5.98, *p* <0.001; men: SMD = −0.66, 95% CI [−0.87, −0.44], z = 6.03, *p* <0.001). Serum UA levels in middle-late stage PD patients with higher H&Y scales were significantly lower than early stage PD patients with lower H&Y scales (SMD = 0.63, 95% CI [0.36,0.89], z = 4.64, *p* <0.001).

**Conclusions:**

Our study showed that the serum UA levels are significantly lower in PD and the level is further decreased as the disease progresses. Thus it might be a potential biomarker to indicate the risk and progression of PD.

## 1 Introduction

Parkinson’s disease (PD) is the second most common neurodegenerative disease that affects approximately 1–2% of the population over 60 years[1]. PD is characterized by an extensive and progressive loss of dopaminergic neurons in the substantia nigra pars compacta[2]. The cause and pathogenesis resulting from the loss of selective dopamine neurons in PD remain elusive. But increasing evidence suggests that an oxidative stress plays an important role in the degeneration of dopaminergic neurons in PD[3]. Consequently, research efforts have been directed towards understanding the pathological role of oxidative stress in the pathogenesis and in the hope of developing more effective therapy approaches for the treatment of PD[4].

Uric acid (UA), one of the end products of purine metabolism is a natural antioxidant in blood and brain tissue, which has neuroprotective effects on PD by effectively scavenging oxygen radicals and reactive nitrogen[5–6]. Numerous large prospective studies have shown that the high levels of serum or plasma urate reduced the risk of PD [7–8]. Recently, the association between serum urate levels and PD has gained intensive interests, but results of these studies have been inconsistent. Some studies have shown a clear trend of lower serum urate levels in PD and subgroups based on sex [9–17], while others showed no significant variation of lower serum urate levels in PD [18–23]. Our previous studies showed that the serum UA levels in PD patients were significantly lower than sex and age-matched healthy controls and the UA concentrations were decreased with the deterioration of the illness[24].

To evaluate the potential relationship between serum urate levels and PD, a meta-analysis was conducted in this present study by combining all available data together.

## 2 Materials and methods

### 2.1 Search strategies

A computerized search of Pubmed, Web of Science, and the Cochrane Library up to Dec 2015 was conducted using the following search strategy: “Parkinson’s disease”, (“blood”, “plasma” or “serum”), and (“uric acid” or “urate”). The search was restricted to English language. The reference lists of relevant studies were also searched for possible studies meeting criteria. If there was an initial disagreement on studies to be included, discussion among researchers established universal agreement. A flowchart of information pertaining to identification, screening, eligibility, and final studies included was constructed according to “Preferred Reporting Items for Systematic Reviews and Meta-analyses” (PRISMA) guidelines [25].

### 2.2 Inclusion and exclusion criteria

Studies comparing serum uric acid in patients with PD and control subjects were included. The inclusion criteria were (1) case-control studies design, only papers showing comparative analysis of patients with PD against healthy controls were considered; (2) all PD patients met the UK Brain Bank diagnostic criteria; and (3) the data of the serum uric acid levels were either available in the report or obtainable from the corresponding author. The exclusion criteria were studies which only included (1) duplicate of earlier publications; (2) papers focused on familial PD and cohort design; and (3) the serum uric acid levels were acquired from in vitro, animal model, and autopsy samples.

### 2.3 Data extraction

Duplicates were deleted and the title and abstract of each article were scanned for relevance independently by two researchers. The full texts of potentially relevant studies were then retrieved and assessed for eligibility by established criteria detailed above.

Data for each individual serum uric acid assessed in eligible studies were extracted into an Excel spreadsheet (author, year of publication, sample size, mean or median, standard deviation or interquartile range, and method). One author (Bo Zhou) extracted all data. The method used to extract data was independently verified by another author (Min Wen). Data were then rechecked for accuracy (Bo Zhou).

### 2.4 Study quality assessment

Each study that was included was assessed independently by two reviewers (Yun Gou and Yun-Hua Chen) using an established form for case-control studies, the Newcastle-Ottawa Scale (NOS)[26], which is used to assess the quality of observational studies. Discrepancies were reported to and settled by a third party (Bo Zhou). Three major aspects of study quality were scored: 1) selection of the study groups (0–4 points); 2) determination of the exposure of interest in the studies (0–3 points); and 3) the quality of the adjustment for confounding variables (0–2 points). A study could be scored as a maximum of one star for each item numbered within the categories of Selection and Exposure, while at most two stars could be allocated to Comparability. A higher score represented improved greater quality of the study methodology. A score equal to or higher than six was considered to indicate high study quality.

### 2.5 Data analysis

All statistical analyses were performed using standardized mean difference (SMD) methodology in Review Manager 5.3.3 and STATA 11.0, with user contribute commands for meta-analyses: metan, heterogi, metainf and metabias[27]. All umol/L uric acid concentrations were converted to conventional units (mg/dl), using a conversion rate of 16.81 (1 mg/dl = 59.48 umol/L). The medians and interquartile range in some studies were converted to means and standard deviation in accordance with the protocol provided by Hozo[28]. Several papers did not report the UA levels in total PD patients and controls, the UA levels in total PD patients and controls were pooled by the subgroup in men and women[29]. We used 95% confidence interval (CI) to gauge the precision of the summary estimates. Overall heterogeneity was assessed using I^2^ and H^2^ statistic[30]. A random-effects model or fixed-effects model was used to calculate pooled SMD in the presence or absence of heterogeneity, respectively. We performed sensitivity analyses to assess the influence of individual studies on the pooled estimate. Publication bias was investigated using funnel plots and Egger’s regression test.

## 3 Results

**Fig 1:**
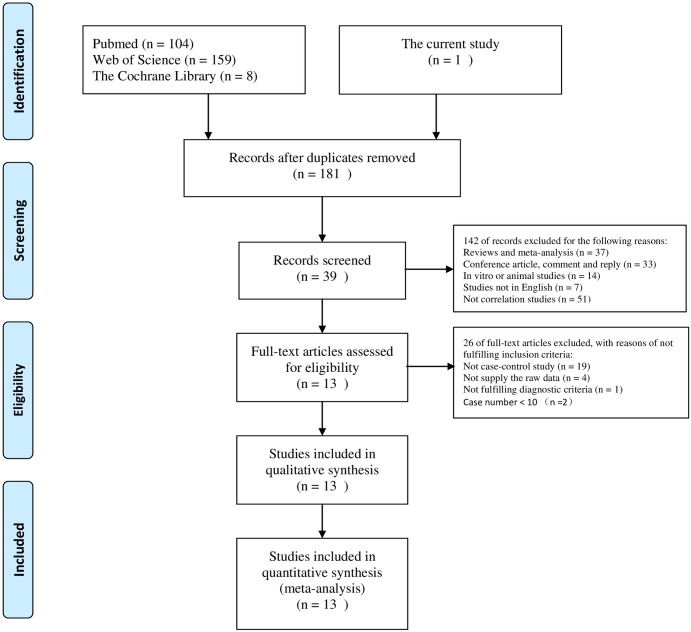
Flow diagram of the study selection process in the meta-analysis.

All studies were identified and the number of studies which were subsequently included or excluded is illustrated as a flow chart (Fig 1). A total of 13 studies, including 2379 patients, met stringent search criteria and were included in the final review. A summary of data extracted from these studies is compiled in Table 1.

**Table 1:** Summary of studies included in the meta-analysis.

No	Author	Year	Country	diagnosed criteria	PD		Control		P value	Ref
					N	UA level	N	UA level		
1	Annanmaki	2007	Finland	UK	37(Total)	278.5±77.4 umol/L	29(Total)	319.7±76.0 umol/L	p = 0.03	[9]
2	Andreadou	2009	Greece	UK	43(Total)	5.47±1.40 mg/dl	47(Total)	6.36±4.3 mg/dl	p = 0.009	[19]
					22(male)	6.16±1.13 mg/dl	25(male)	7.19±2.1 mg/dl	p = 0.058	
					21(Female)	4.63±1.16 mg/dl	22(Female)	5.43±1.58 mg/dl	p = 0.08	
3	Ikeda	2011	Japan	UK	119(Total)	4.42±1.3 mg/dl	120(Total)	5.55±2.06 mg/dl		[10]
					56(male)	4.9±1.5 mg/dl	60(male)	6.2±2.4 mg/dl	p<0.01	
					63(Female)	4.0±0.9 mg/dl	60(Female)	4.9±1.4 mg/dl	p<0.01	
4	Maetzler	2011	Germany	UK	55(Total)	5.29±1.33mg/dl	76(Total)	5.49±1.87 mg/dl		[11]
					29(male)	5.3(4.6–7.8) mg/dl	27(male)	6.3(3.7–10.1) mg/dl	p<0.01	
					26(Female)	4.2(2.8–7.9) mg/dl	49(Female)	5.1(1.9–7.4) mg/dl	p<0.05	
5	Annanmaki	2011	Finland	UK	28(Total)	284.5±73.0 umol/L	12(Total)	309.1±87.5 umol/L	p>0.05	[20]
6	Sun	2012	Chinese	UK	411(Total)	243.38±78.91 umol/L	396(Total)	282.97±90.80 umol/L	p<0.01	[12]
					207(male)	252.62±87.75 umol/L	202(male)	295.14±84.18 umol/L	p<0.01	
					204(Female)	234.00±67.73 umol/L	194(Female)	270.30±95.80 umol/L	p<0.01	
7	Zhang	2012	Chinese	UK	534(Total)	278.10±87.31 umol/L	614(Total)	348.17±86.25 umol/L	p<0.001	[13]
					341(male)	294.60±89.21 umol/L	384(male)	376.00±82.55 umol/L	p<0.01	
					193(Female)	248.95±75.11 umol/L	230(Female)	301.71±71.05 umol/L	p<0.01	
8	Jesus	2013	Spain	UK	161(Total)	4.68±1.66 mg/dl	178(Total)	5.37±1.60 mg/dl	p<0.001	[14]
					90(male)	5.23±1.38 mg/dl	108(male)	5.82±1.61 mg/dl	p<0.05	
					71(Female)	3.99±1.50 mg/dl	70(Female)	4.68±1.31 mg/dl	p<0.05	
9	Gonzalez-Aramburu	2013	Spain	UK	365(Total)	5.23±1.43 mg/dl	132(Total)	5.28±1.77 mg/dl		[22]
					209(male)	5.61±1.37 mg/dl	42(male)	6.27±1.96 mg/dl	p = 0.04	
					156(Female)	4.73±1.36 mg/dl	90(Female)	4.82±1.47 mg/dl	p = 0.09	
10	Kim	2014	Pusan	UK	30(Total)	4.7±1.4 mg/dl	25(Total)	5.7±1.5 mg/dl	p = 0.001	[15]
					19(male)	5.3±1.4 mg/dl	12(male)	6.7±1.3 mg/dl	p = 0.018	
					11(Female)	3.8±1.0 mg/dl	13(Female)	4.8±1.2 mg/dl	p = 0.011	
11	Lolekha	2015	Thailand	UK	100(Total)	5.01±1.46 mg/dl	100(Total)	5.54±1.29 mg/dl	p = 0.007	[23]
					50(male)	5.79±1.21 mg/dl	50(male)	6.20±1.22 mg/dl	p = 0.093	
					50(Female)	4.24±1.26 mg/dl	50(Female)	4.89±1.01 mg/dl	p = 0.005	
12	Qin	2015	Chinese	UK	425(Total)	241.26±59.45 umol/L	459(Total)	300.09±60.06 umol/L	p<0.001	[17]
					282(male)	254.97±55.80 umol/L	297(male)	321.37±53.89 umol/L	p<0.05	
					143(Female)	215.11±58.00 umol/L	162(Female)	260.96±50.70 umol/L	p<0.05	
13	Current group		Chinese	UK	71(Total)	265.78±58.55 umol/L	79(Total)	299.04±77.46 umol/L	p<0.05	[24]
					38(male)	294.92±56.70 umol/L	42(male)	331.54±70.34 umol/L	p<0.05	
					33(Female)	232.23±40.16 umol/L	37(Female)	262.15±68.81 umol/L	p<0.05	

### 3.1 Results of quality assessment

Quality assessment of the individual studies showed that the NOS score ranged from six to eight. These results indicated that the quality of methodology was generally good (Table 2).

**Table 2:** Results of quality assessment.

No	Author	Selection	Exposure	Comparability	Total score
1	Annanmaki	4	3	1	8
2	Andreadou	3	3	1	7
3	Ikeda	3	3	1	7
4	Maetzler	4	3	1	8
5	Annanmaki	4	3	1	7
6	Sun	4	3	1	8
7	Zhang	3	3	1	7
8	Jesus	3	3	1	7
9	Gonzalez-Aramburu	4	3	1	8
10	Kim	3	3	1	7
11	Lolekha	4	3	1	8
12	Qin	4	3	1	8
13	Current group	4	3	1	8

### 3.2 Serum uric acid in PD versus control group

**Fig 2:**
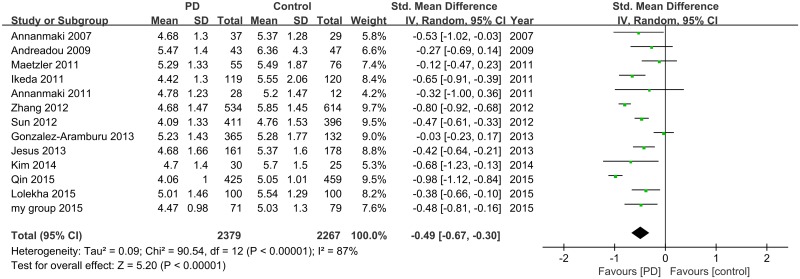
Forest plot of serum uric acid levels in PD compared to control.

13 studies (included 2379 PD and 2267 controls) were used in this analysis. These studies had significant heterogeneity (I^2^ = 87%, 95% CI: [79%, 92%];H^2^ = 7.29, 95% CI: [4.84, 12.25]), so a random-effect model was used to calculate pooled SMD. Forest plot (Fig 2) showed that the diamond was on the left side of the vertical line and did not intersect with the line, which demonstrates a significantly lower concentration of serum uric acid in patients with PD compared to control (SMD: -0.49, 95% CI: [-0.67, -0.30], *Z* = 5.20, *P* < 0.001).

### 3.3 Analysis of serum uric acid in PD versus control in different geographic regions

**Fig 3:**
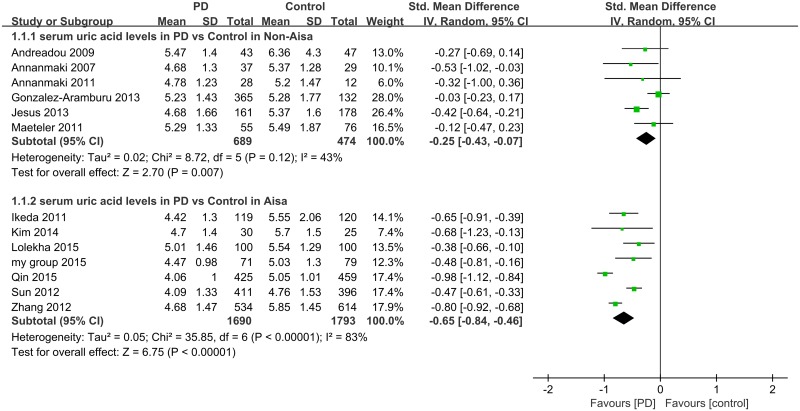
Forest plot of serum uric acid levels in PD compared to control in Asia and Non-Asia groups.

In total, 1690 patients with PD and 1793 healthy controls were included in Asia group and 689 patients with PD and 474 healthy controls were included in Non-Asia subgroup. Forest plot (Fig 3) showed that the diamond was on the left side of the vertical line and did not intersect with the line, which demonstrates a significantly lower mean concentration of serum uric acid in patients with PD compared to healthy controls in Asia (I^2^ = 83%, 95% CI: [67%, 92%]; H^2^ = 5.76, 95% CI: [2.89, 11.56], SMD = −0.65, 95% CI [−0.84, −0.46], Z = 6.75, *p*<0.001) and Non-Asia (I^2^ = 43%, 95% CI: [0%, 77%]; H^2^ = 1.69, 95% CI: [1.00, 4.41], SMD = −0.25, 95% CI [−0.43, −0.07], Z = 2.70, *p* = 0.007). In addition, according to the present results this association was relatively more significant in Asia group than in Non-Asia group.

### 3.4 Sex subgroup analysis of serum uric acid in PD versus control

**Fig 4:**
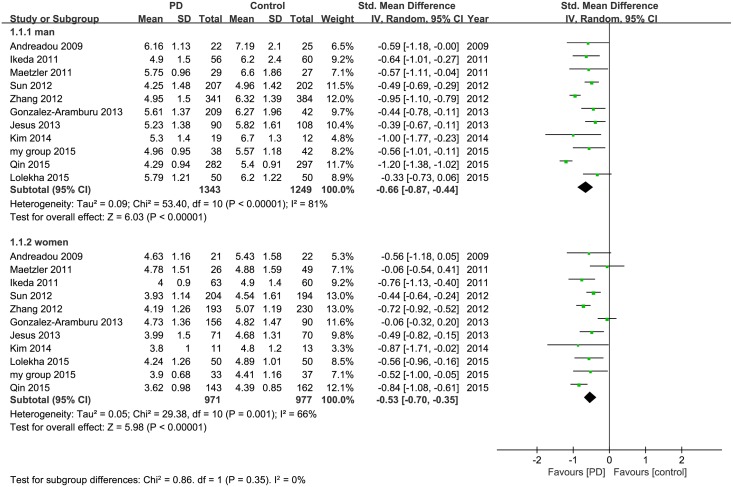
Forest plot of serum uric acid levels in PD compared to control in men and women.

In total, there were 971 patients with PD and 977 healthy controls in women subgroup and 1343 patients with PD and 1249 healthy controls were included in men subgroup. The meta-analysis results indicated lower uric acid levels in patients with PD compared to healthy controls for women (I^2^ = 66%, 95% CI: [36%, 82%]; H^2^ = 2.89, 95% CI: [1.44, 5.76], SMD = −0.53, 95% CI [−0.70, −0.35], z = 5.98, *p*<0.001). Similar results were also obtained for men (I^2^ = 81%, 95% CI: [68%, 89%]; H^2^ = 5.29, 95% CI: [3.24, 9.0], SMD = −0.66, 95% CI [−0.87, −0.44], z = 6.03, *p*<0.001). (Fig 4)

### 3.5 Analysis of serum uric acid in subgroups based on severity of PD

**Fig 5:**
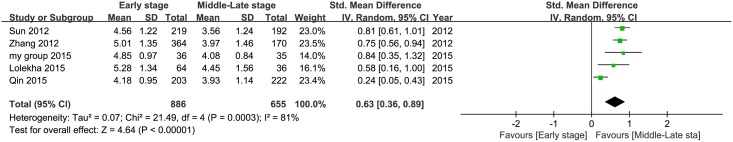
Forest plot of serum uric acid levels in early stage compared to middle-late stage PD.

To explore the effect of disease severity on the uric acid levels in patients with PD, H&Y scales subgroup analysis was performed. Since the H&Y staging criteria was inconsistent in these studies (Qin’s and zhang’s study according to the old H&Y stages criteria, the others according to the modified criteria), we divided PD patients into two groups of early and middle-late stage in order to incorporate more data (early stage, H&Y scale < = 2.5; middle-late stage, H&Y scale >2.5). Five studies (886 early stage and 655 middle-late stage patients of PD) were included in this analysis. A summary of data extracted from these studies is compiled in Table 3. These studies had significant heterogeneity (I^2^ = 81%, 95% CI: [57%, 92%]; H^2^ = 5.29, 95% CI: [2.25, 12.25]). The heterogeneity is mainly derived from the different standards of H&Y stages. The meta-analysis results indicated lower uric acid levels in patients with middle-late stage than early stage (SMD = 0.63, 95% CI [0.36,0.89], z = 4.64, p<0.001) (Fig 5).

**Table 3:** Summary of studies included in the H&Y scale subgroup analysis.

No	Author	Year	Country	Diagnosed criteria	Early stage PD		Middle-Late stage PD		Ref
					N	UA level	N	UA level	
1	Sun	2012	China	UK	219	4.56±1.22	192	3.56±1.24	[12]
2	Zhang	2012	China	UK	364	5.01±1.35	170	3.97±1.46	[13]
3	Qin	2015	China	UK	203	4.18±0.95	222	3.93±1.14	[17]
4	Lolekha	2015	Thailand	UK	64	5.28±1.34	36	4.45±1.56	[23]
5	Current group	2015	China	UK	36	4.85±0.97	35	4.08±0.84	[24]

### 3.6 Publication bias and sensitivity analysis

No publication bias was detected by Egger’s regression test and funnel plot. Sensitivity analysis suggested that the results were stable and reliable (S1–S18 Figs).

## 4 Discussion

Oxidative damage has been implicated as a core contributor to the neurodegenerative process in PD[3]. Floor et al. showed an increased level of oxidative stress in postmortem brain of PD compared to controls[31]. The link between oxidative stress and dopaminergic neuronal degeneration is further supported by modeling the motor aspects of PD in animals with toxins that cause oxidative stress including 1-methyl-4-phenyl-1,2,3,6-tetrahydropyridine (MPTP), rotenone, 1,1’-dimethyl-4,4’-bipyridinium dichloride (paraquat), and 6-hydroxydopamine (6-OHDA)[32]. Urate is one of the main end products of purine metabolism and is present in the brain and body fluids[33]. Urate is a natural antioxidant and iron chelator, thus it may play an important neuroprotective role in PD by its antioxidant and iron chelator activities[34–35]. Cipriani et al. showed that an administration of UA in experimental models of PD was found to suppress oxidative stress and prevent against nigral cell death[34]. Guerreiro et al. reported that the urate exhibits antioxidant properties and ameliorates human dopaminergic cell damage in cell culture following exposure to pesticides or iron ions[36], and can reduce the death of dopaminergic neurons in primary midbrain cultures[37]. Chen et al. observed increased urate concentrations in the brain tissue of urate oxidase KO mice which attenuated toxic effects of 6-OHDA on nigral dopaminergic neurons. Conversely, overexpression of urate oxidase has reduced brain urate concentrations and exacerbates the lesions of the dopaminergic neurons[38]. Hence urate has been considered as a potential therapeutic target of an antioxidant and iron chelator.

For the past few years, much attention has been paid to the relationship of uric acid in PD. A growing number of studies have correlated higher serum UA levels with both a lower risk of developing PD and a slower rate of disease progression. However, the results from previous studies are inconsistent. So a meta-analysis was conducted in this present study to evaluate the potential relationship between serum urate levels and PD.

Our results from the meta analysis indicated that the serum UA levels in PD patients were significantly lower than sex and age-matched healthy controls. Subgroup analysis showed that the results had no geographic regional or sex differences. In order to investigate the relationship between the serum UA levels and the disease progression, the PD patients were divided into two subgroups according to their H&Y scales. Data showed that the middle-late stage PD patients with higher H&Y scales (> = 2.5) had significantly lower serum UA levels than early stage PD patients with lower H&Y scales (<2.5). We could not conduct a meta-analysis to compare the serum UA levels between before and after the treatment, because of very few available publications, and only data from Andreadou’s paper[19] were included in our analysis. This paper reported that the lower serum UA levels were observed in treated patients compared to the untreated. So the research about the serum UA levels in before and after the treatment should be further strengthened in the future.

Many factors might contribute to the discordance of the results of serum UA levels in PD: the number of recruited studies and sample size in each study was relatively limited; these studies did not exclude the influence of genetic factors (such as Uric acid transport gene mutated)[39] and lifestyle factors (diet, diuretics and alcohol consumption) [40], which could alter UA levels in blood; heterogeneity of patients, such as age, disease duration, disease severity, diagnosis criteria and treatment status. Most of the studies did not report the H&Y scale, and the standard of H&Y scale was not unified. These factors could influence the results of relationship between serum UA levels and progression of PD.

Compared to the previous reports[41], present study had following advantages: the most recent literature was included, this is the first meta-analysis study to explore the connection of serum UA levels and the progression of PD and the regional differences of serum uric acid in PD versus control subjects. However, our research also had some limitations. One is the Publication biases, which is a fallibility factor of meta-analysis. Publication bias were not found in our study by funnel plots and Egger’s regression test, but the power of both methods was lower for meta-analyses based on 10 or fewer studies[42]; the other limitation was the higher heterogeneity, which can greatly affect conclusions of meta-analysis. However all statistical tests for heterogeneity are currently weak[43]. In order to solve this problem, sensitivity analysis (Kontopantelis et al. [44]) was adopted. Our sensitivity analysis showed that the higher heterogeneity has no effect on our results.

In conclusion, our results show that the serum UA levels are significantly lower in PD and UA concentrations unceasingly decrease with the deterioration of the illness. These findings suggest that the serum UA levels might be a potential biomarker to indicate the risk and progression of PD.

## Supporting information

S1 Fig Funnel plots of serum uric acid levels in PD compared to control. (TIF)

S2 Fig Egger’s regression test of serum uric acid levels in PD compared to control. (TIF)

S3 Fig Sensitivity analyses of serum uric acid levels in PD compared to control. (TIF)

S4 Fig Funnel plots of serum uric acid levels in PD compared to control in Asia. (TIF)

S5 Fig Egger’s regression test of serum uric acid levels in PD compared to control in Asia. (TIF)

S6 Fig Sensitivity analyses of serum uric acid levels in PD compared to control in Asia. (TIF)

S7 Fig Funnel plots of serum uric acid levels in PD compared to control in Non-Asia. (TIF)

S8 Fig Egger’s regression test of of serum uric acid levels in PD compared to control in Non-Asia. (TIF)

S9 Fig Sensitivity analyses of serum uric acid levels in PD compared to control in Non-Asia. (TIF)

S10 Fig Funnel plots of serum uric acid levels in PD compared to control in man. (TIF)

S11 Fig Egger’s regression test of serum uric acid levels in PD compared to control in man. (TIF)

S12 Fig Sensitivity analyses of serum uric acid levels in PD compared to control in man. (TIF)

S13 Fig Funnel plots of serum uric acid levels in PD compared to control in woman. (TIF)

S14 Fig Egger’s regression test of serum uric acid levels in PD compared to control in women. (TIF)

S15 Fig Sensitivity analyses of serum uric acid levels in PD compared to control in woman. (TIF)

S16 Fig Funnel plots of serum uric acid levels in early stage compared to middle-late stage PD. (TIF)

S17 Fig Egger’s regression test of serum uric acid levels in early stage compared to middle-late stage PD. (TIF)

S18 Fig Sensitivity analyses of serum uric acid levels in early stage compared to middle-late stage PD. (TIF)

S1 File PRISMA checklist. (DOC)
