# Supplementary figures and images for: Serum uric acid levels in patients with Parkinson’s disease: A meta-analysis
Source: PLoS One. 2017 Mar 20;12(3):e0173731. doi: 10.1371/journal.pone.0173731 (PMC5358777; doi:10.1371/journal.pone.0173731)

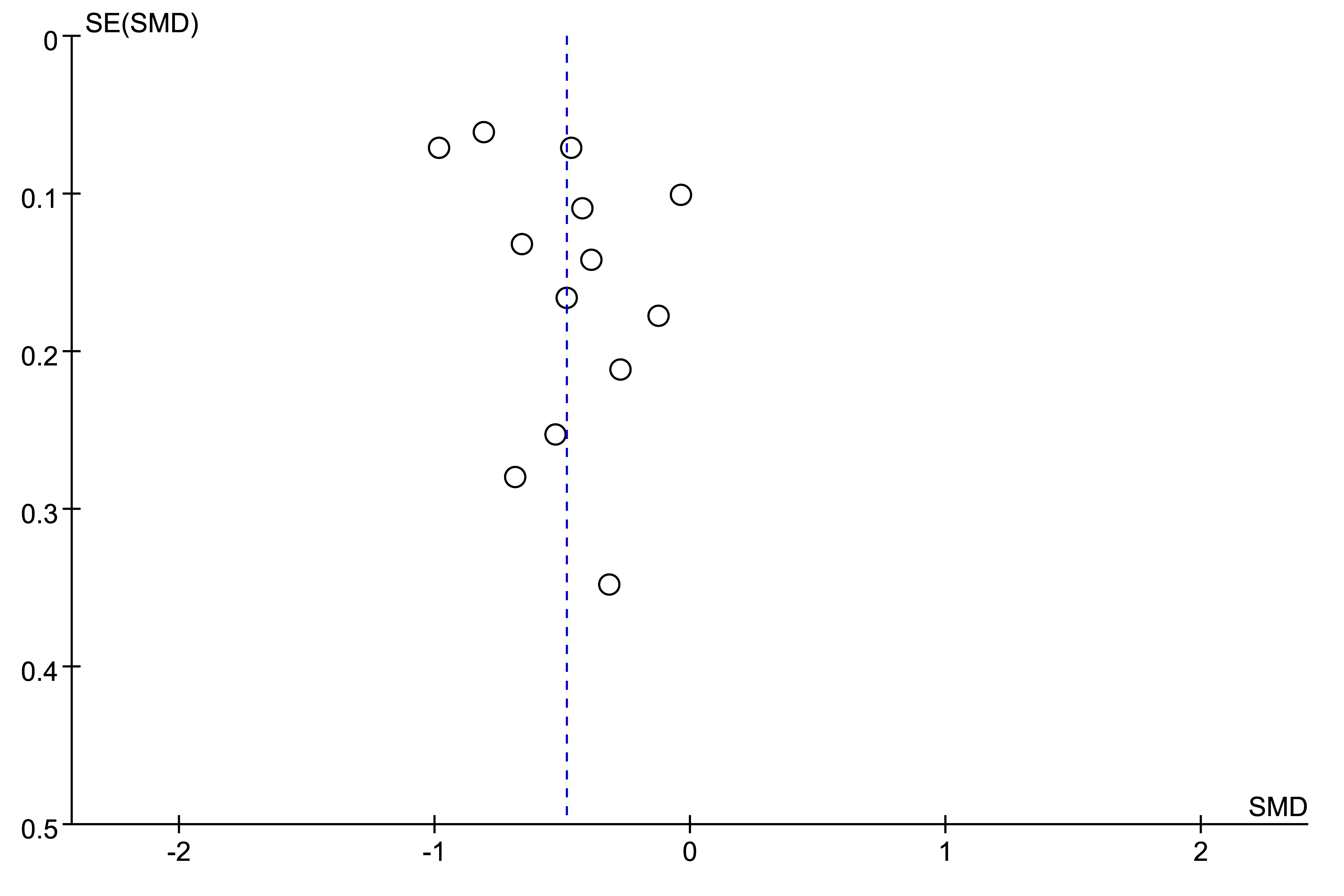

Supplement: S1 Fig — (TIF) [file pone.0173731.s001.tif]

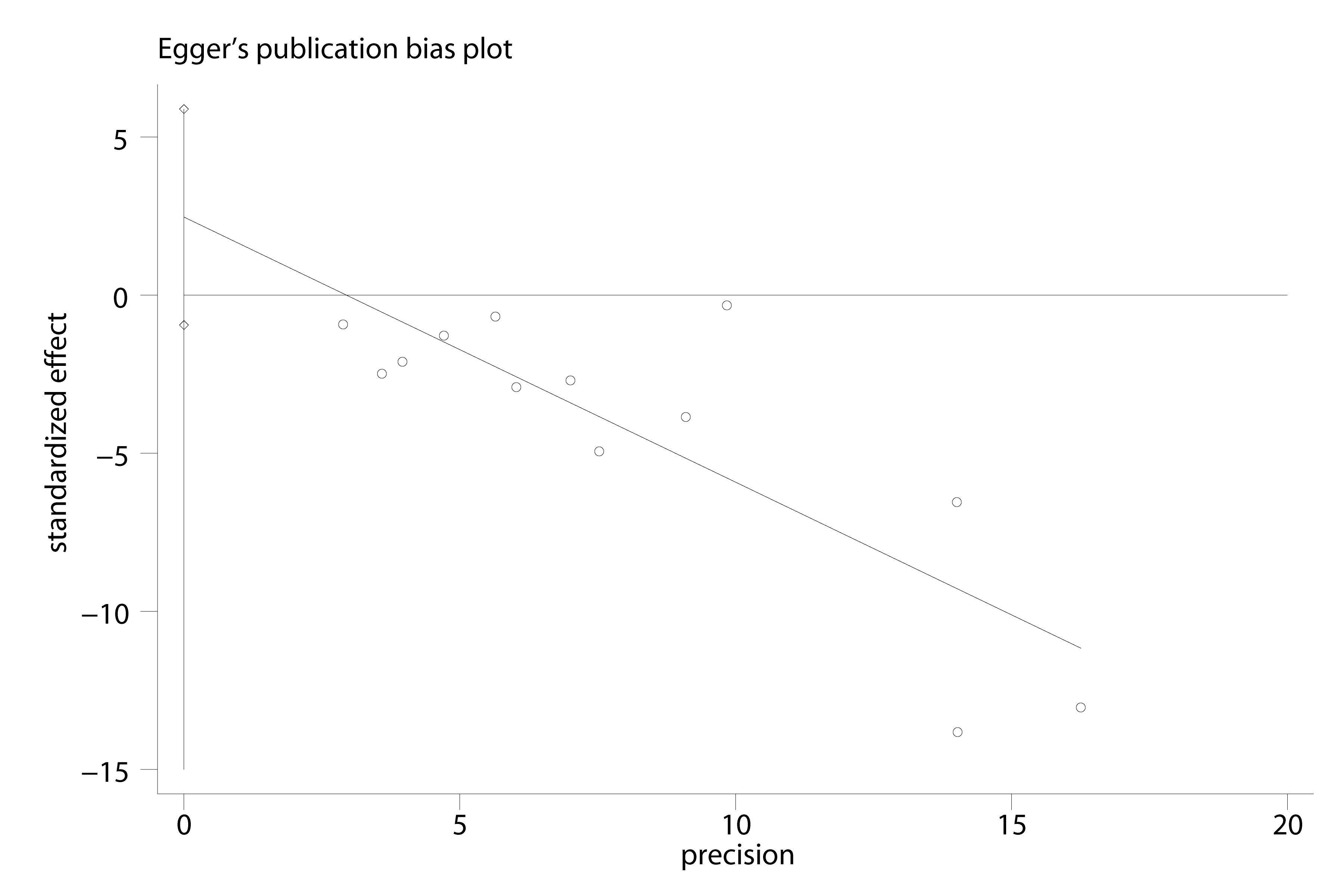

Supplement: S2 Fig — (TIF) [file pone.0173731.s002.tif]

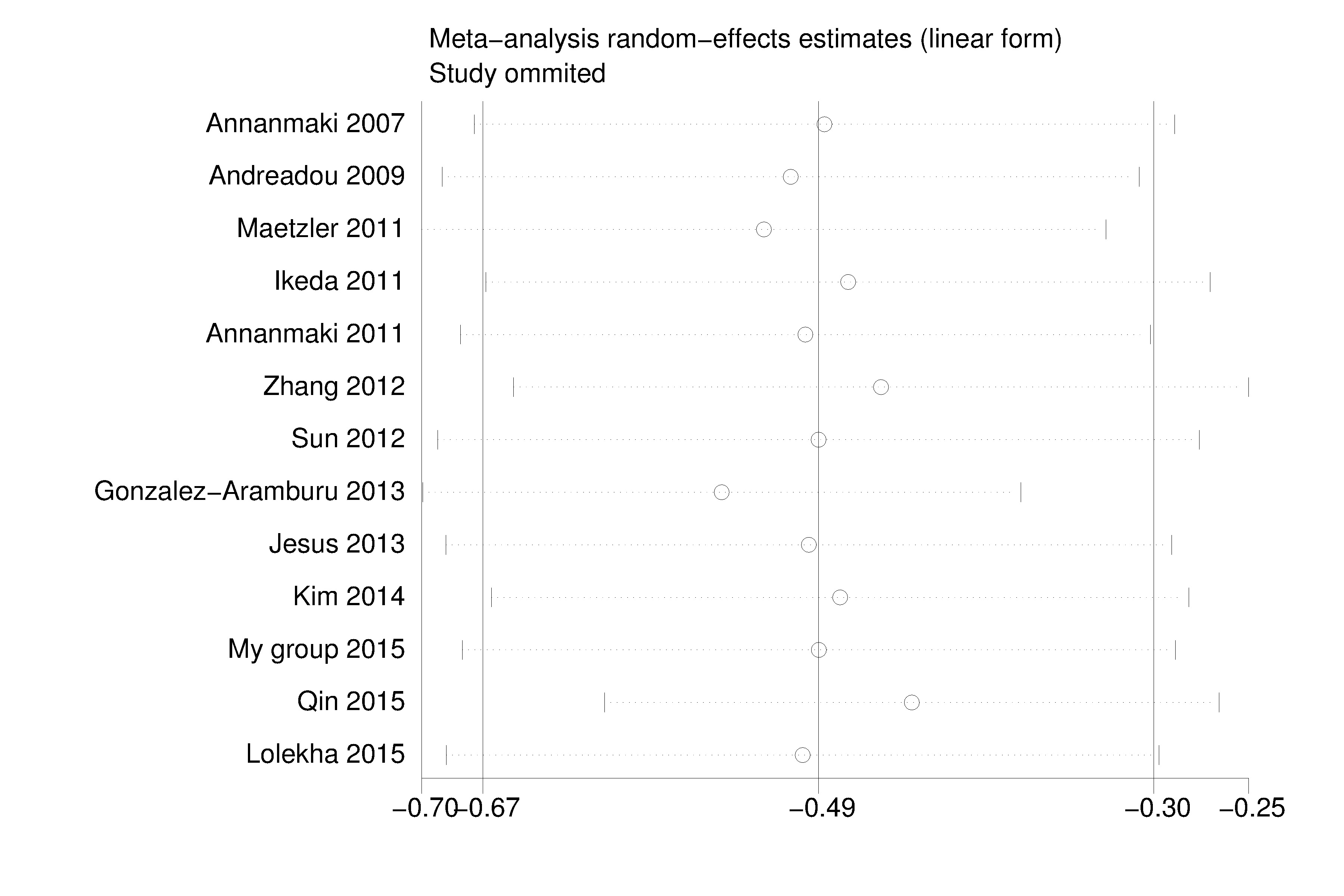

Supplement: S3 Fig — (TIF) [file pone.0173731.s003.tif]

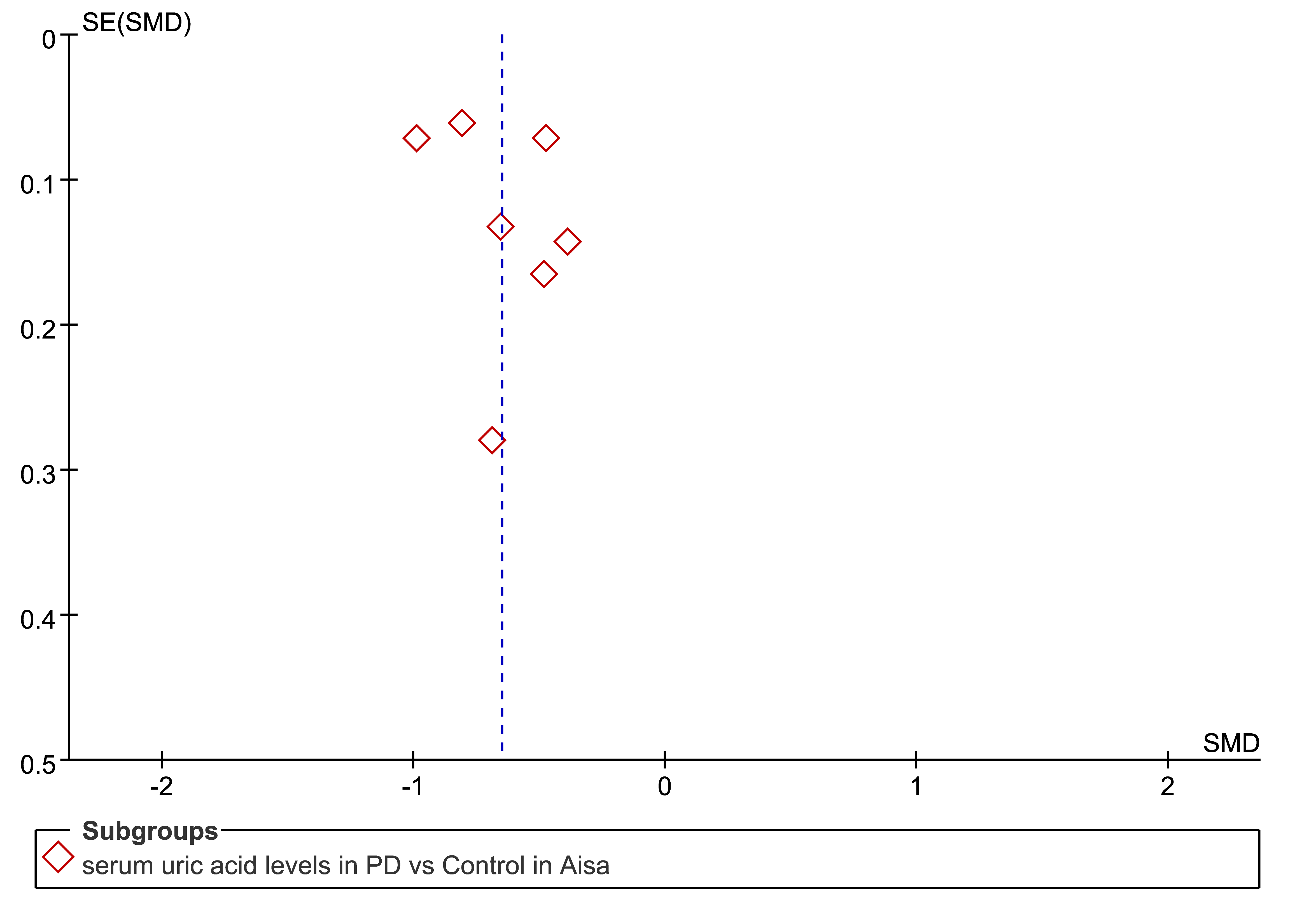

Supplement: S4 Fig — (TIF) [file pone.0173731.s004.tif]

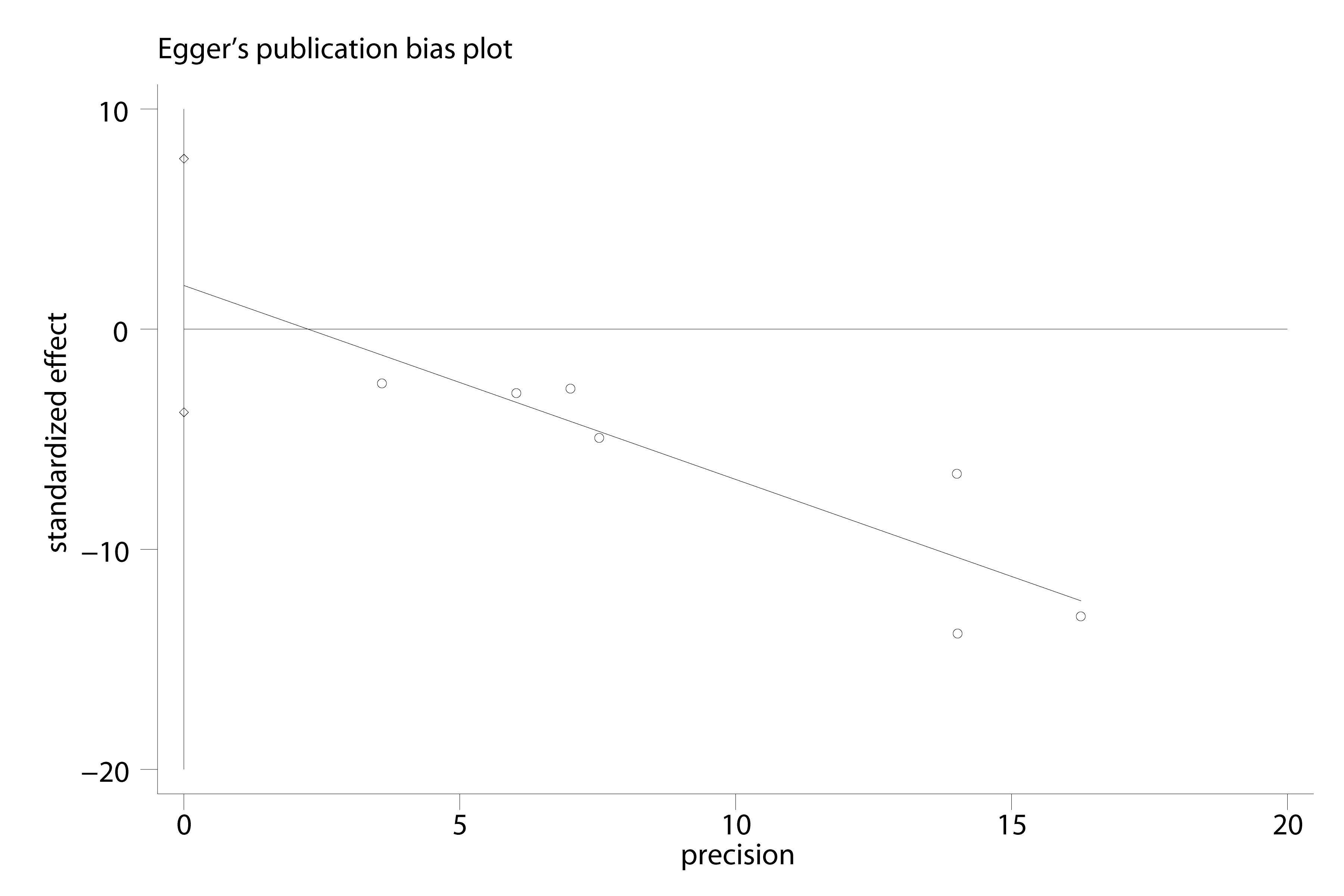

Supplement: S5 Fig — (TIF) [file pone.0173731.s005.tif]

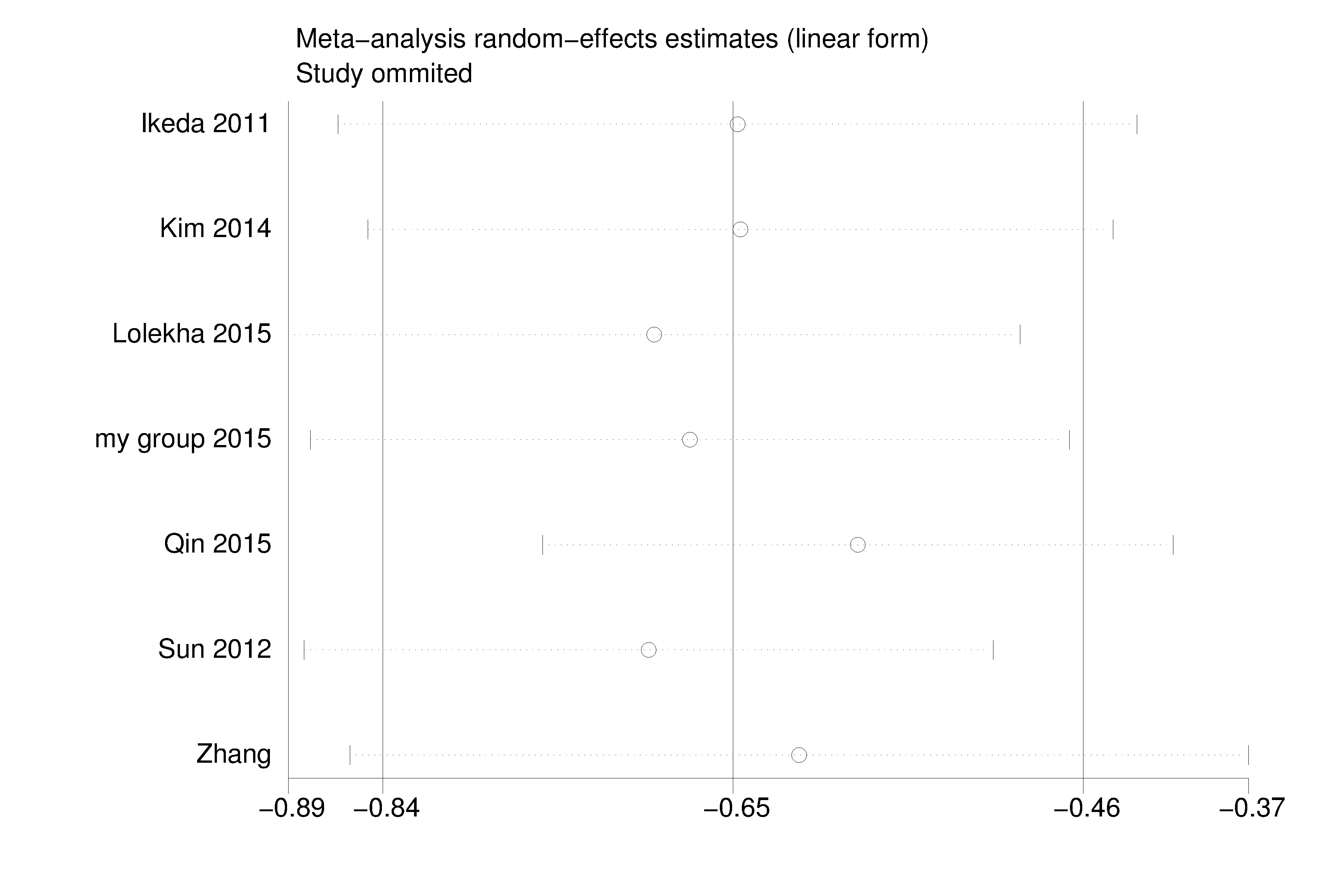

Supplement: S6 Fig — (TIF) [file pone.0173731.s006.tif]

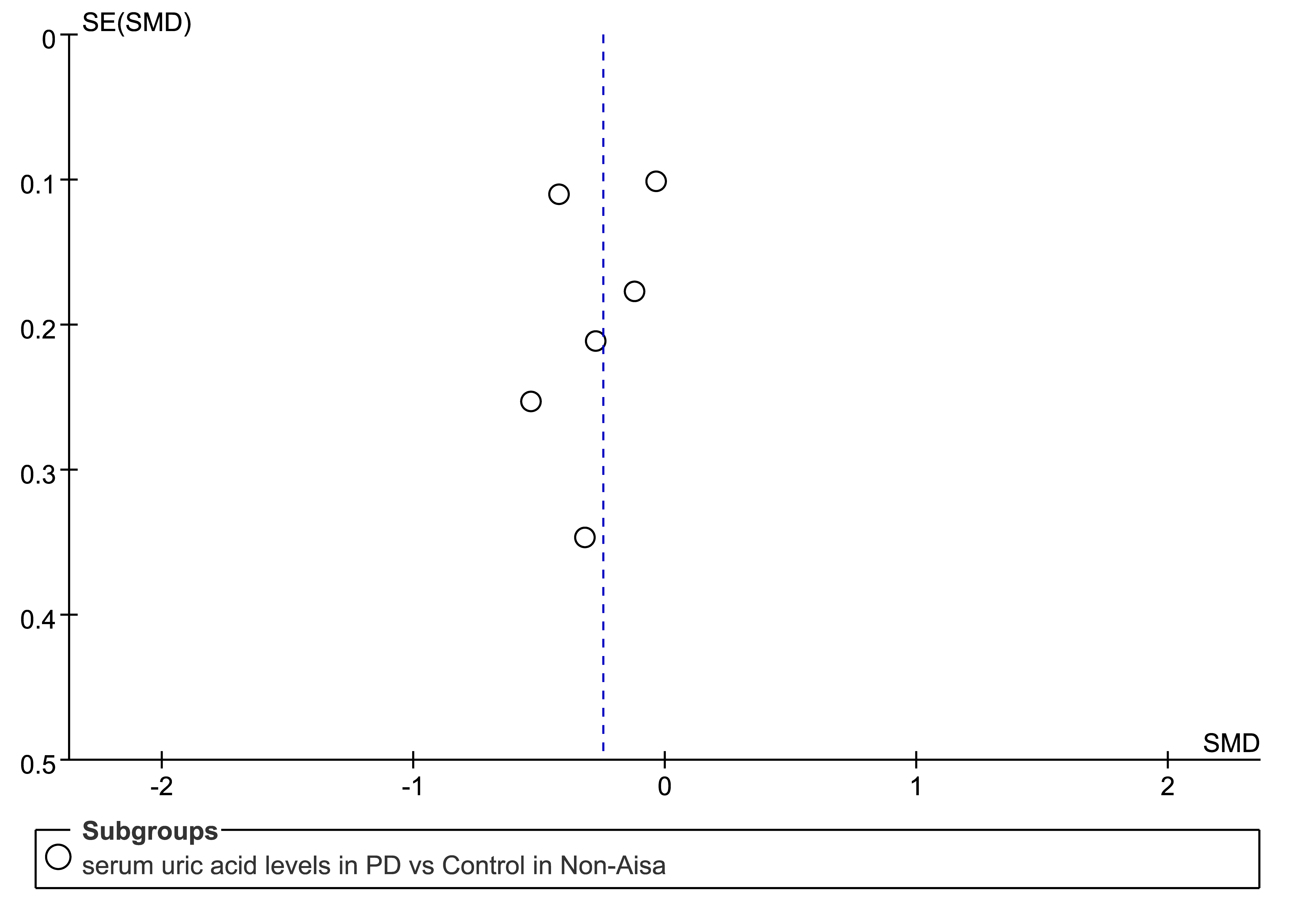

Supplement: S7 Fig — (TIF) [file pone.0173731.s007.tif]

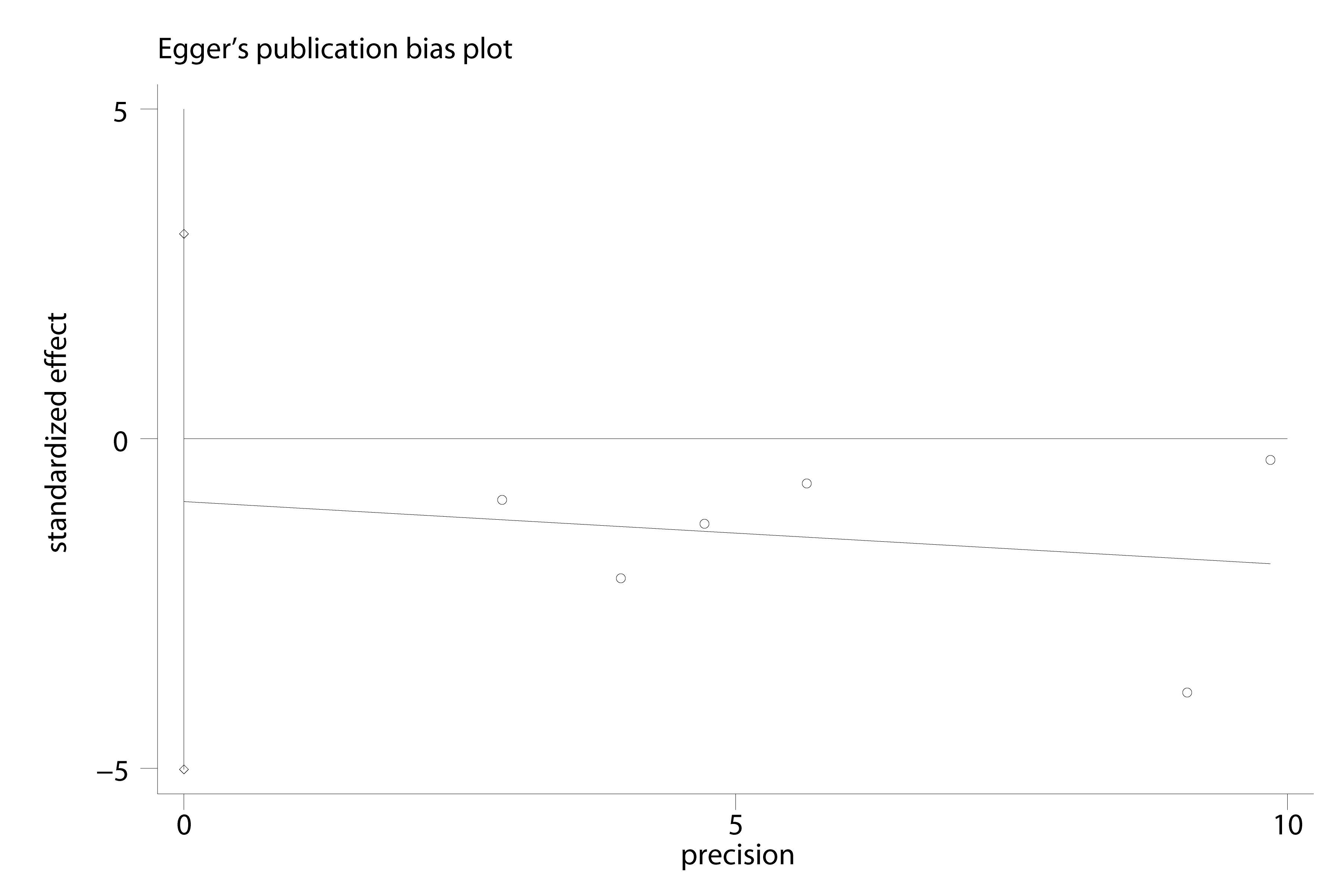

Supplement: S8 Fig — (TIF) [file pone.0173731.s008.tif]

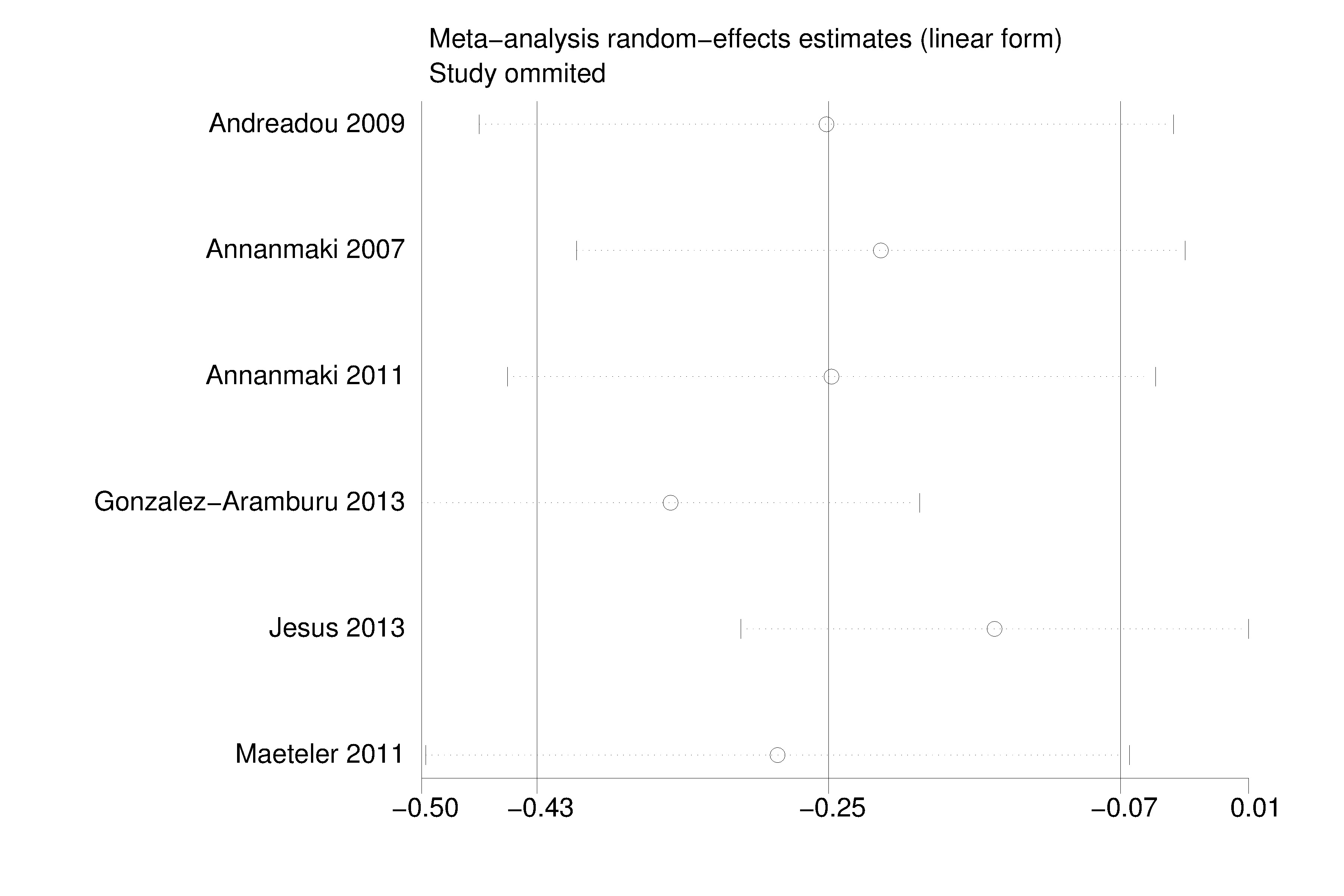

Supplement: S9 Fig — (TIF) [file pone.0173731.s009.tif]

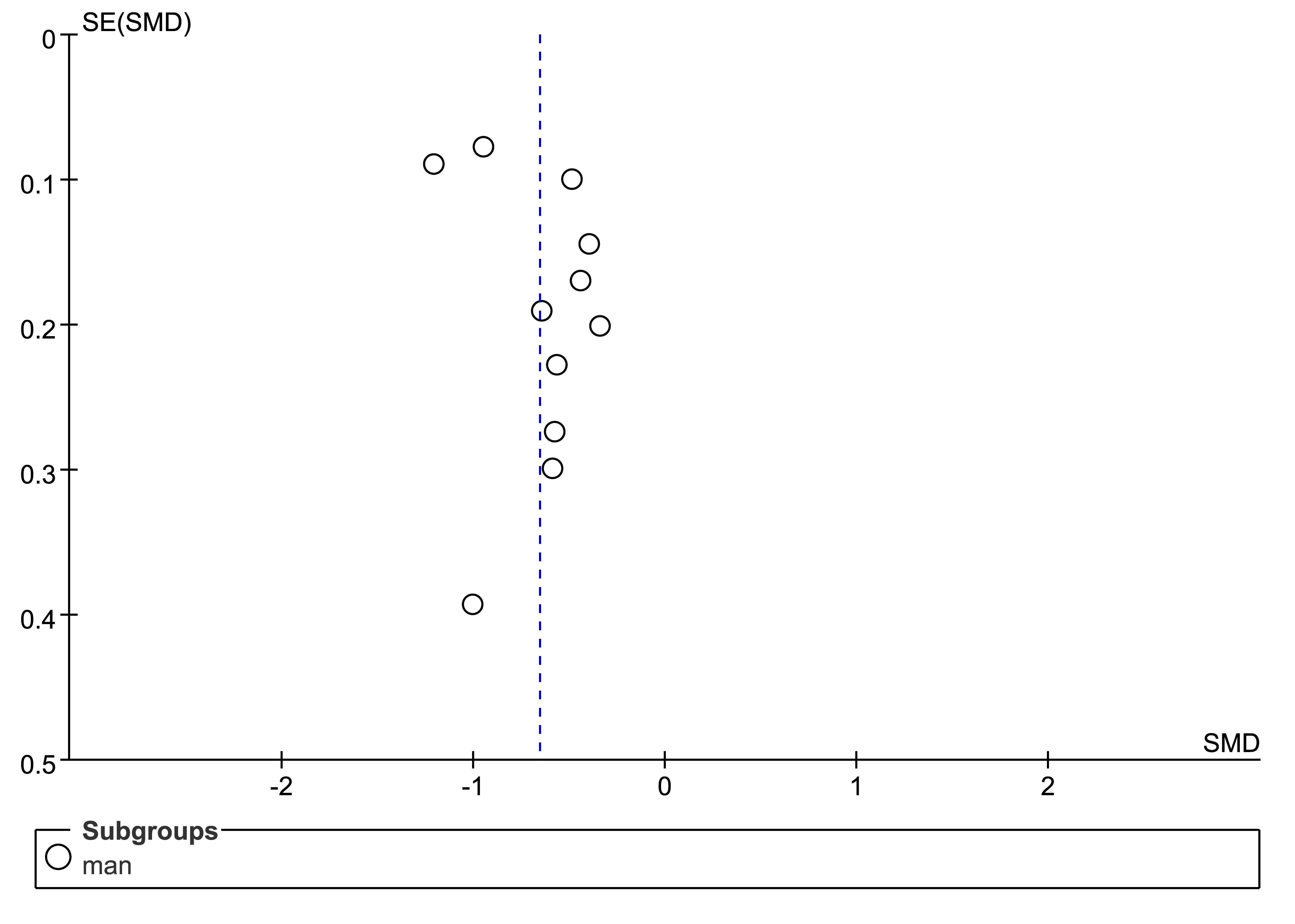

Supplement: S10 Fig — (TIF) [file pone.0173731.s010.tif]

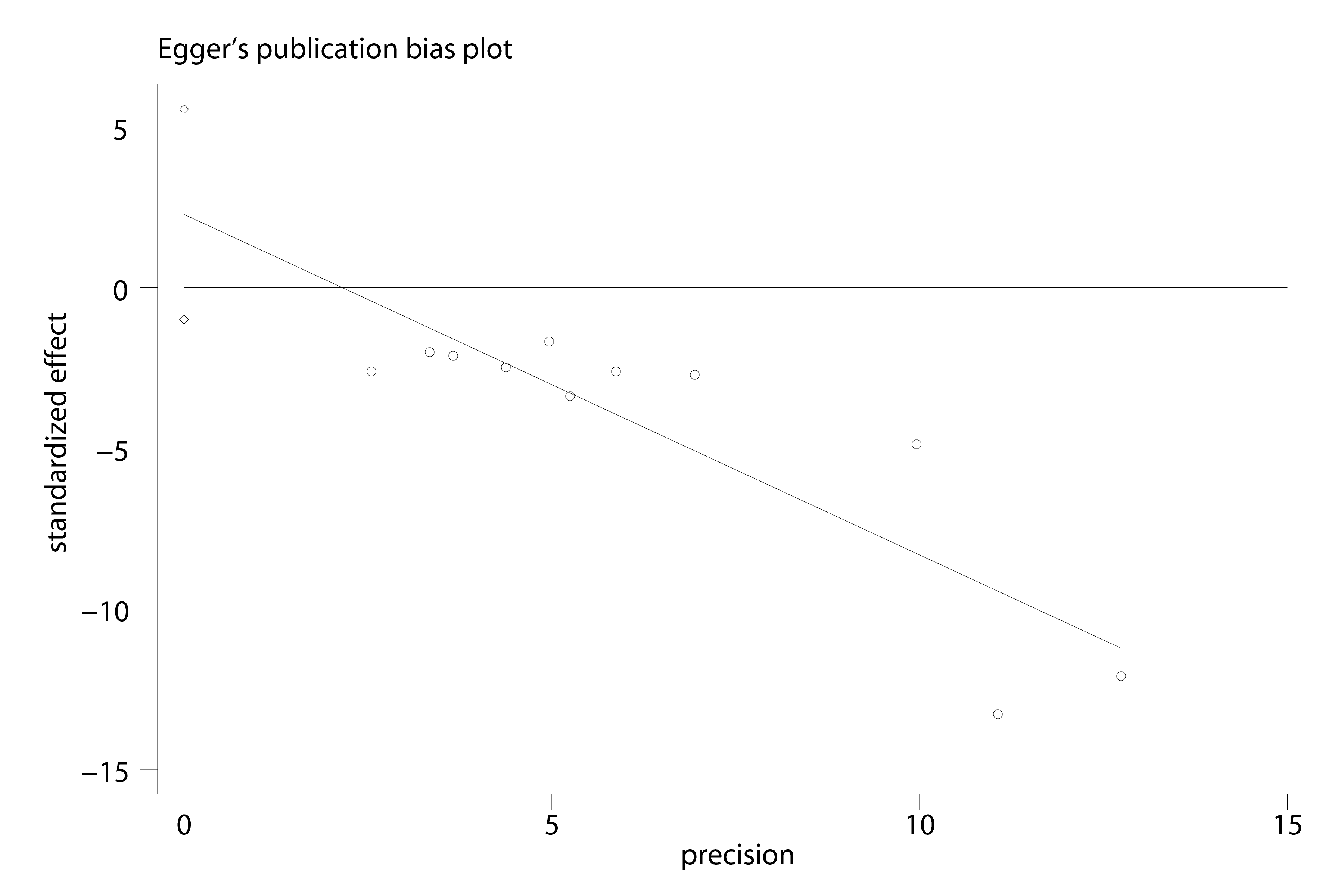

Supplement: S11 Fig — (TIF) [file pone.0173731.s011.tif]

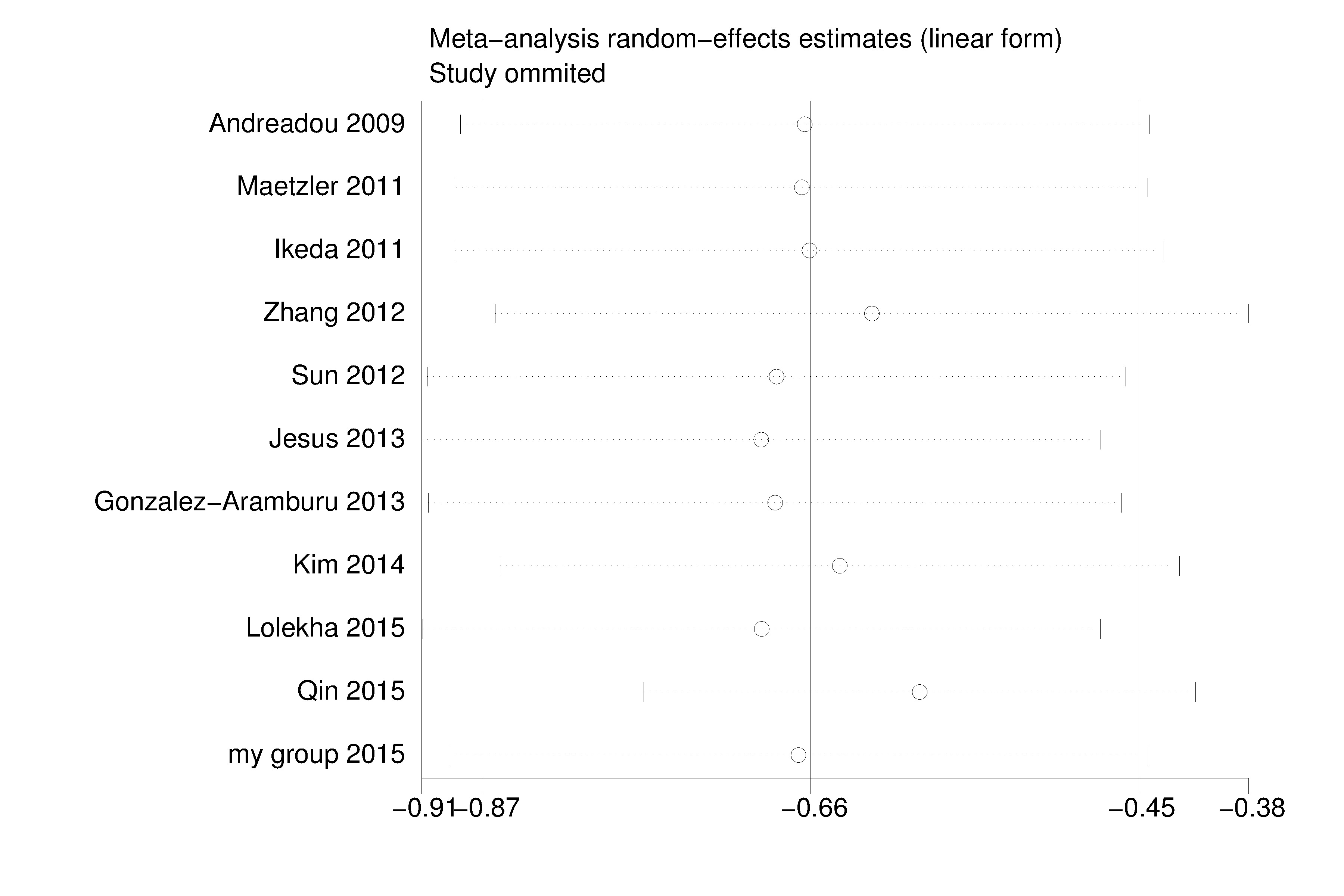

Supplement: S12 Fig — (TIF) [file pone.0173731.s012.tif]

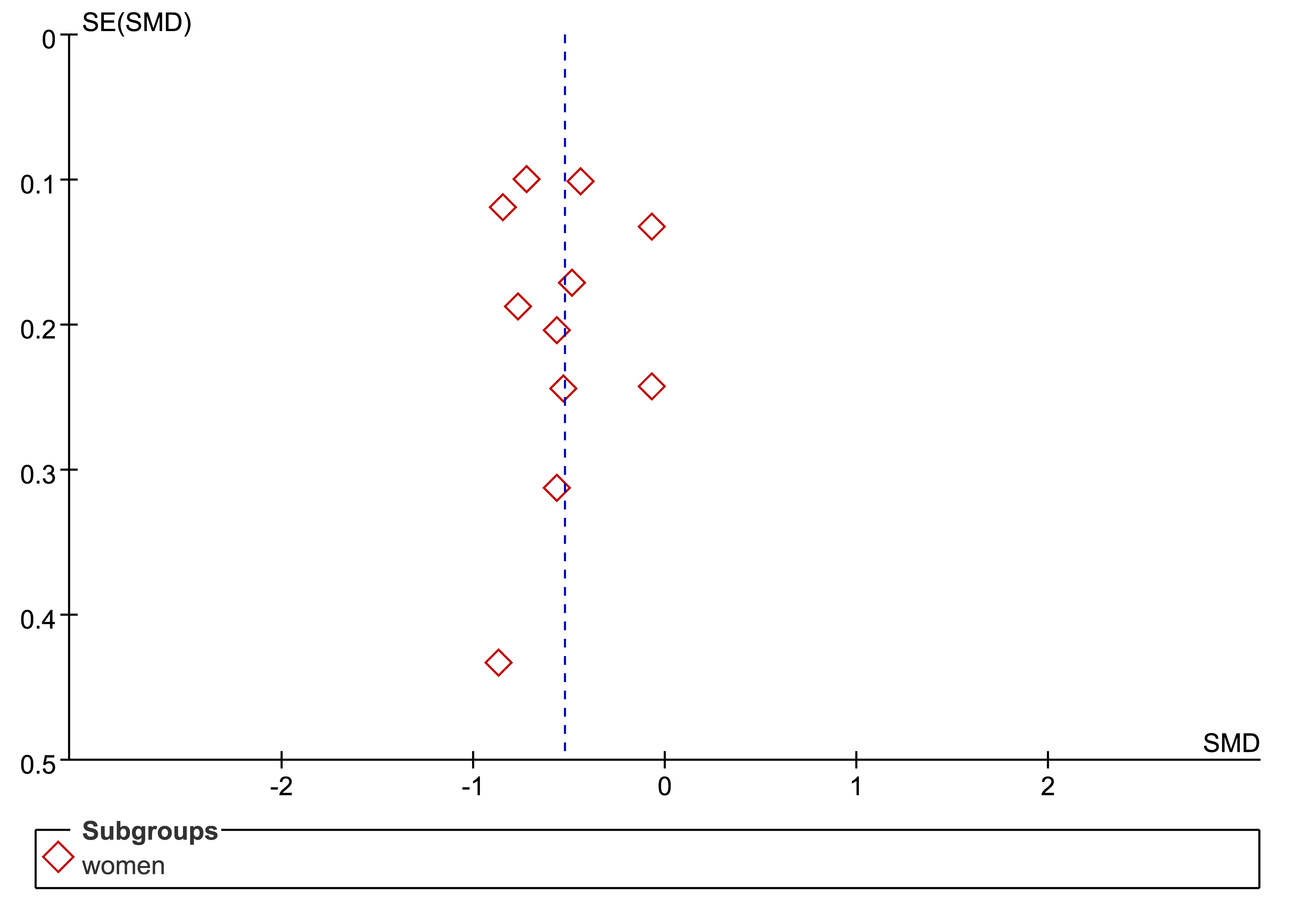

Supplement: S13 Fig — (TIF) [file pone.0173731.s013.tif]

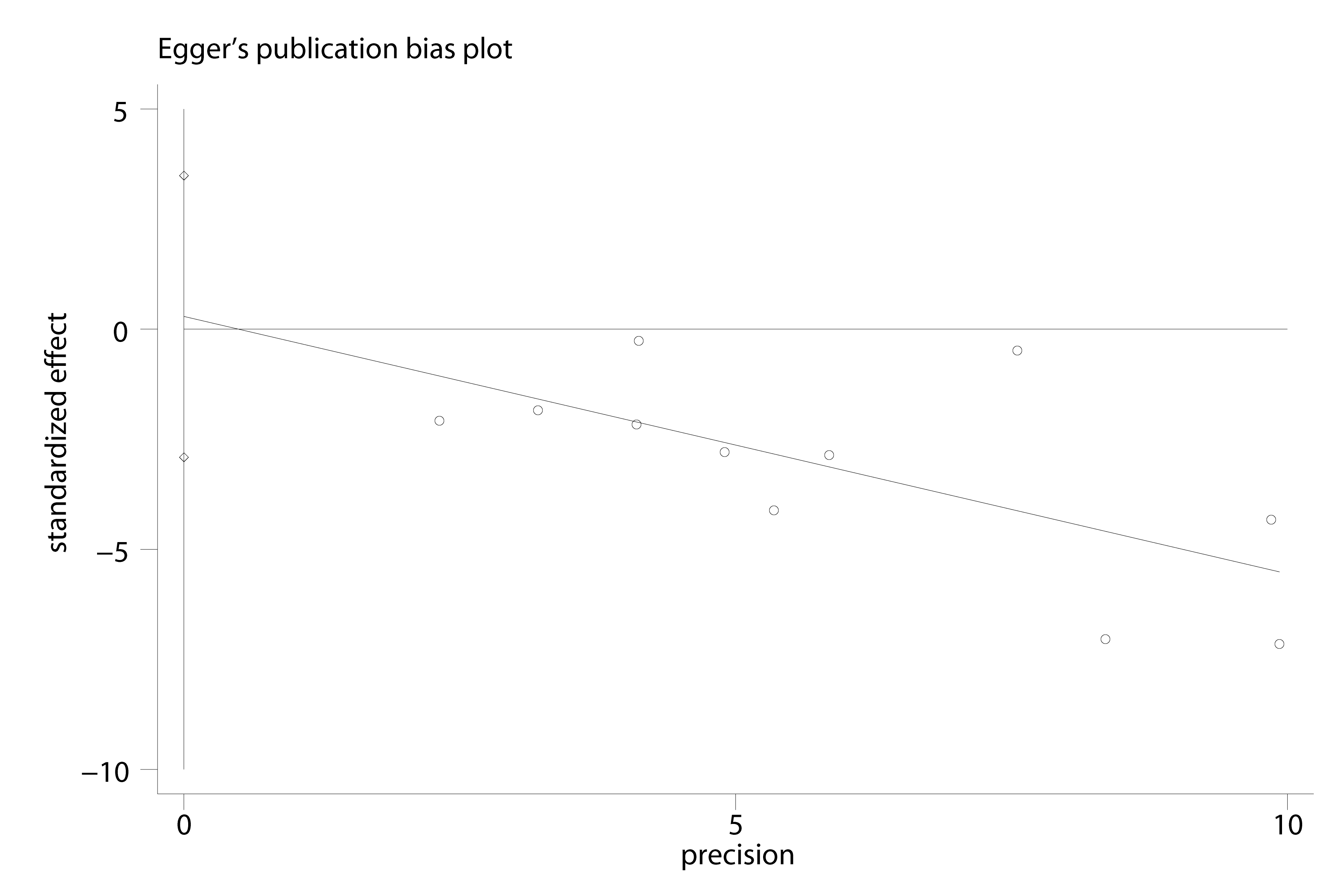

Supplement: S14 Fig — (TIF) [file pone.0173731.s014.tif]

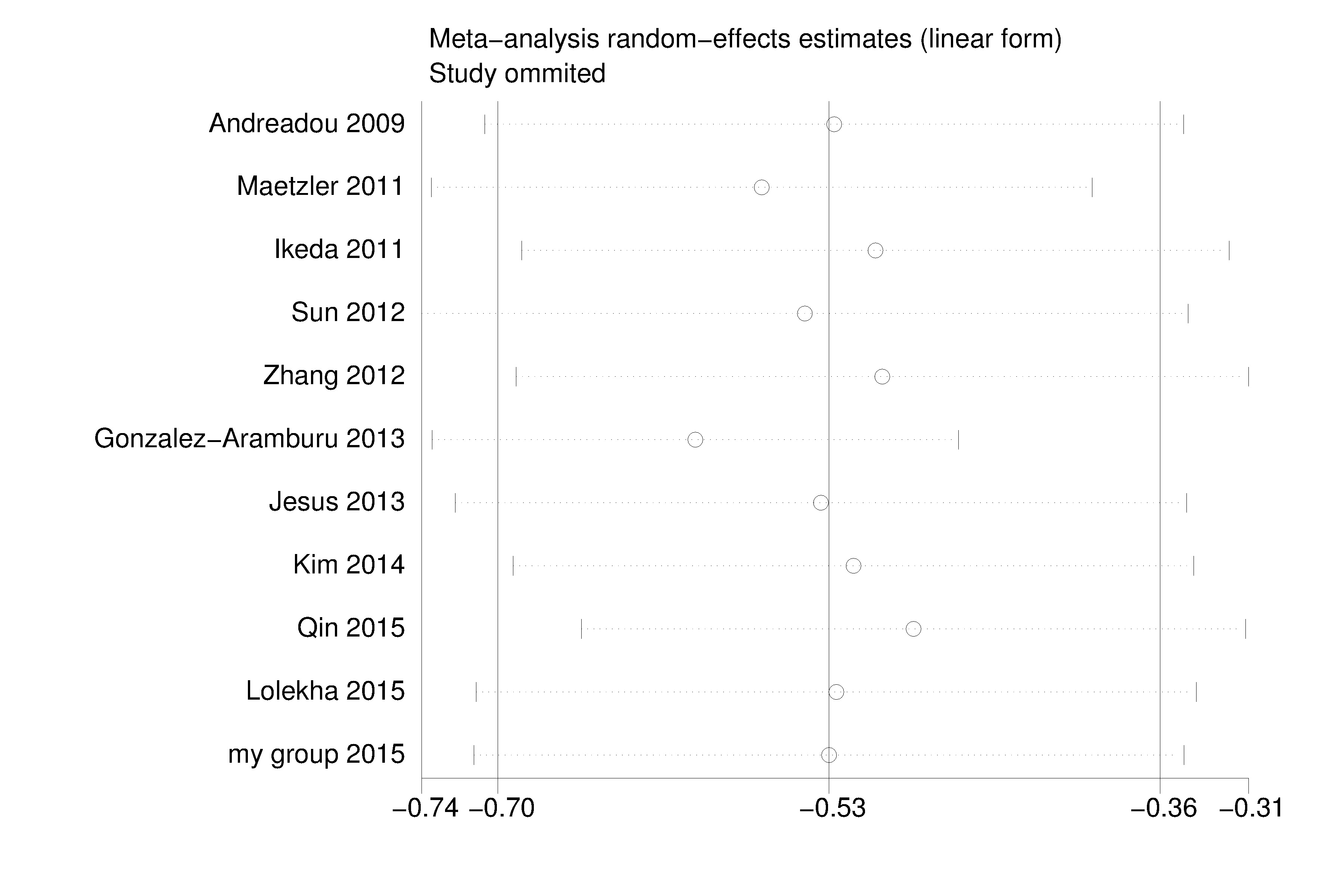

Supplement: S15 Fig — (TIF) [file pone.0173731.s015.tif]

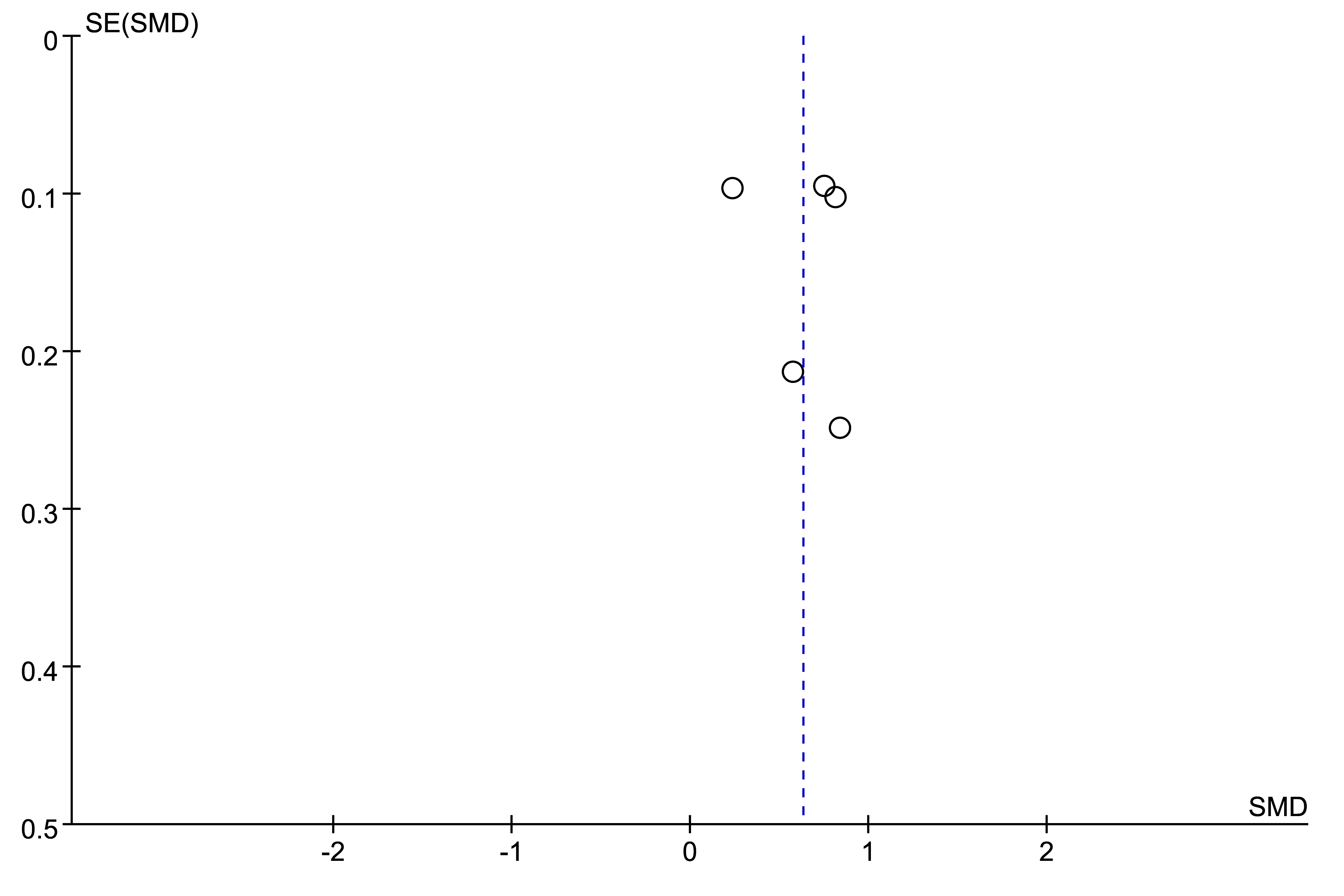

Supplement: S16 Fig — (TIF) [file pone.0173731.s016.tif]

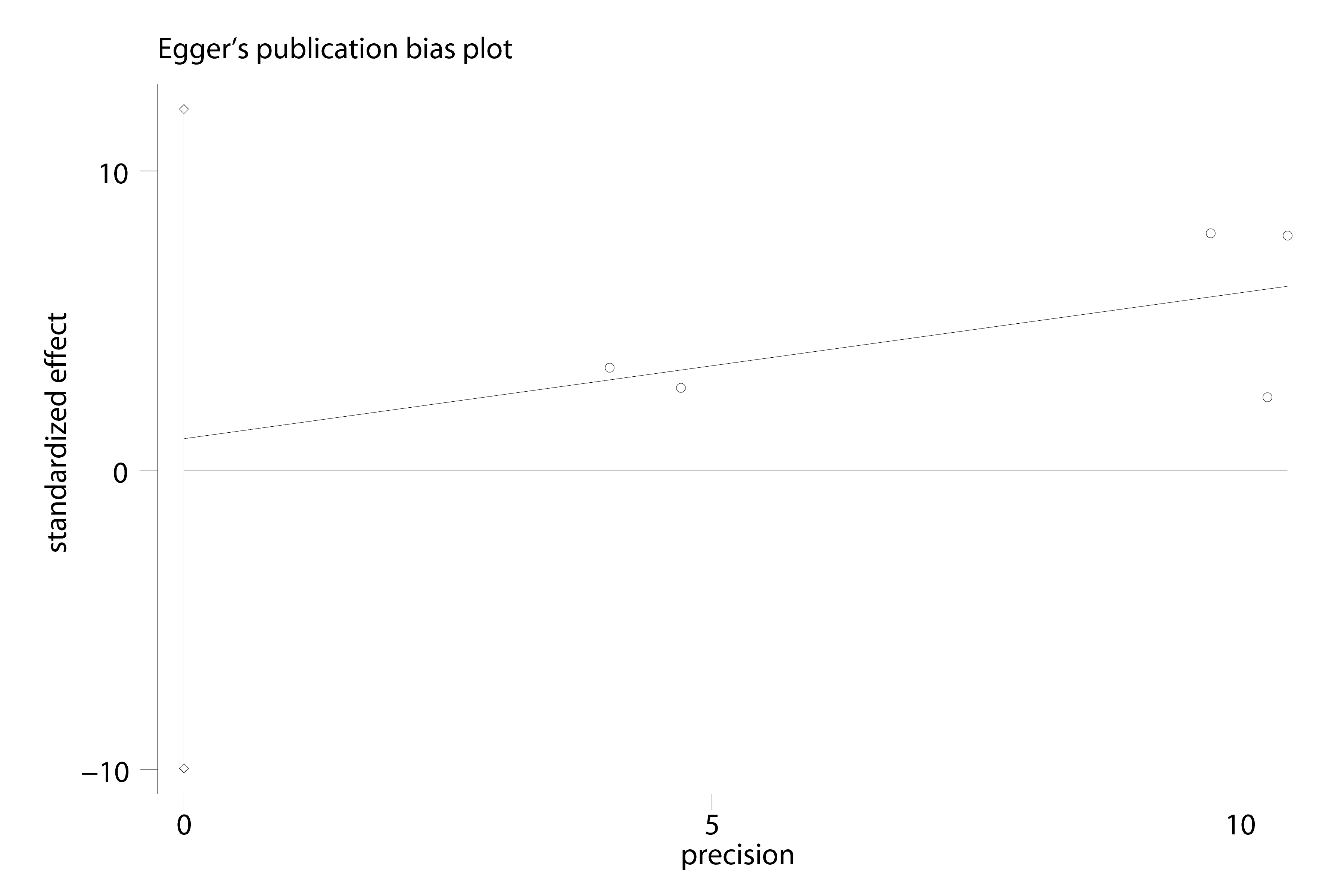

Supplement: S17 Fig — (TIF) [file pone.0173731.s017.tif]

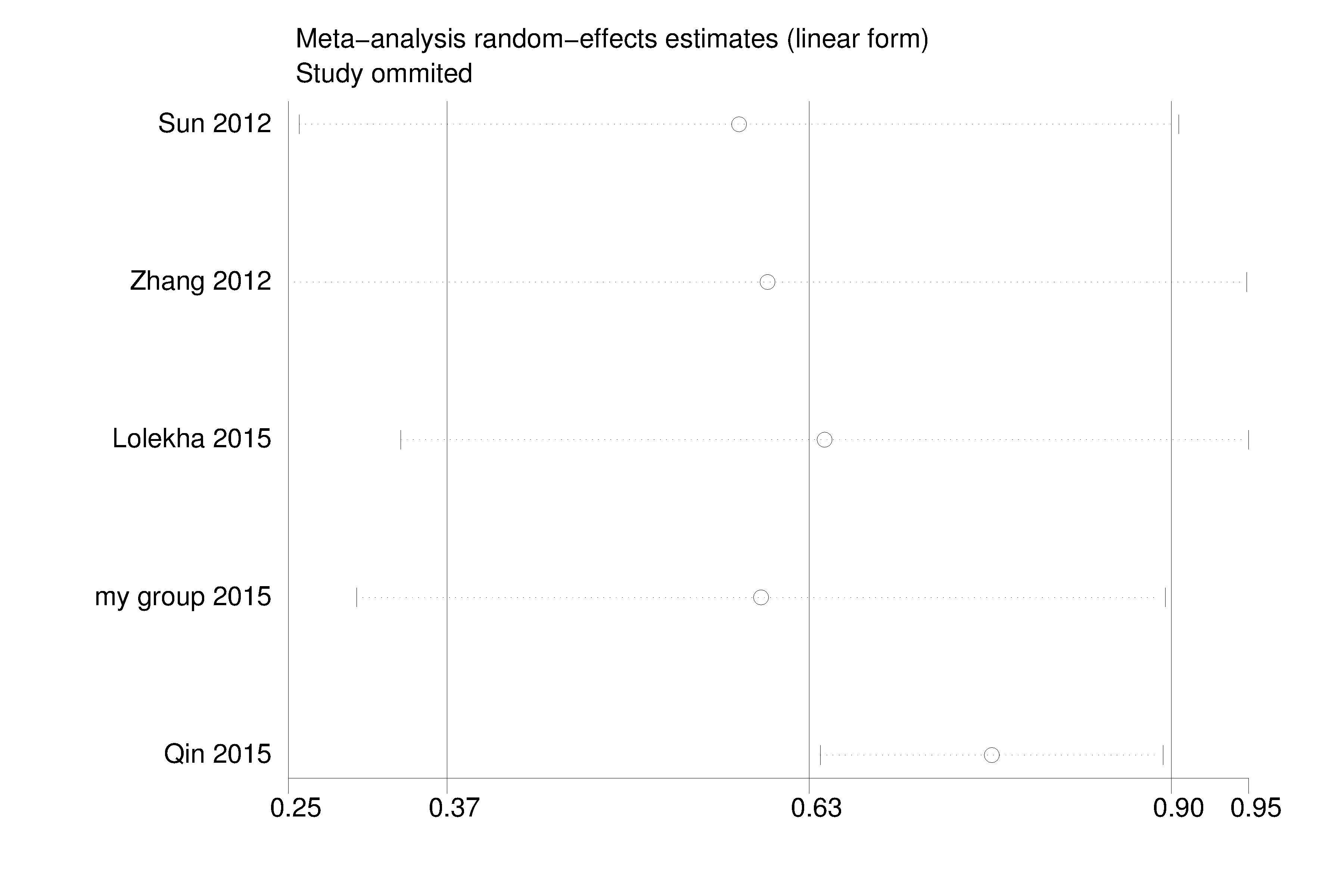

Supplement: S18 Fig — (TIF) [file pone.0173731.s018.tif]
